# Double‐Dipole Induced by Incorporating Nitrogen‐Bromine Hybrid Cathode Interlayers Leads to Suppressed Current Leakage and Enhanced Charge Extraction in Non‐Fullerene Organic Solar Cells

**DOI:** 10.1002/advs.202302460

**Published:** 2023-07-03

**Authors:** Yangchao Zheng, Jingjing Zhao, Huanpeng Liang, Zhenmin Zhao, Zhipeng Kan

**Affiliations:** ^1^ Center on Nanoenergy Research Guangxi Colleges and Universities Key Laboratory of Blue Energy and Systems Integration Carbon Peak and Neutrality Science and Technology Development Institute School of Physical Science & Technology Guangxi University Nanning 530004 China; ^2^ State Key Laboratory of Featured Metal Materials and Life‐cycle Safety for Composite Structures Nanning 530004 China

**Keywords:** cathode interlayer, charge extraction, double‐dipole, fill factor

## Abstract

The cathode interlayer plays a vital role in organic solar cells, which can modify the work function of electrodes, lower the electron extraction barriers, smooth the surface of the active layer, and remove solvent residuals. However, the development of organic cathode interlayer lags behind the rapidly improved organic solar cells because their intrinsic high surface tension can lead to poor contact with the active layers. Herein, a double‐dipole strategy is proposed to enhance the properties of organic cathode interlayers, which is induced by incorporating nitrogen‐ and bromine‐containing interlayer materials. To verify this approach, the state‐of‐the‐art active layer composed of PM6:Y6 and two prototypical cathode interlayer materials, PDIN and PFN‐Br is selected. Using the cathode interlayer PDIN: PFN‐Br (0.9:0.1, in wt.%) in the devices can reduce the electrode work function, suppress the dark current leakage, and improve charge extractions, leading to enhanced short circuit current density and fill factor. The bromine ions tend to break from PFN‐Br and form a new chemical bond with the silver electrode, which can adsorb extra dipoles directed from the interlayer to silver. These findings on the double‐dipole strategy provide insights into the hybrid cathode interlayers for efficient non‐fullerene organic solar cells.

## Graphical abstract

Incorporating bromine‐ and nitrogen‐containing electron transport materials can induce double interfacial dipoles, lowering the silver electrode's work function, decreasing the charge extraction barriers, and suppressing the dark current leakage. This approach practical is confirmed by using multiple nitrogen‐bromine hybrid cathode interlayer combinations and prove this method can constantly improve the power conversion efficiency in organic solar cells composed of PM6:Y6.

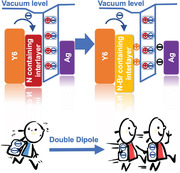

## 1 Introduction

Organic solar cells (OSCs) have attracted significant research interest and achieved remarkable power conversion efficiency (PCE).^[^ ^1^, ^2^, ^3^, ^4^, ^5^, ^6^ ^]^ Especially with the development of non‐fullerene acceptors (NFAs), a PCE of over 19% has been realized.^[^ ^7^, ^8^, ^9^ ^]^ Therefore, in conventional devices, cathode interlayers were usually deposited between the low‐function metal electrode and the active layer to ensure efficient electron extraction. Solution‐processed organic cathode interlayers have drawn considerable attention in this case due to the complex thermal evaporation method and poor interfacial contact between inorganic interlayers and the active layer.^[^ ^10^, ^11^, ^12^ ^]^

Owing to the extended planar structure of the perylene diimides (PDI) and naphthalene diimides (NDI) units, a set of cathode interlayers with PDI and NDI core, such as PDIN, PDINO, PDINN, NDI‐N, PNDIT‐F3N, and NDI‐NBr was designed.^[^ ^13^, ^14^, ^15^, ^16^ ^]^ While these materials were used as top cathode interlayers, an interfacial dipole with the positive end pointing to the electrode and the negative end pointing to the active layer could form, enhancing the built‐in field and the charge carrier transport. For example, using PDINN in the OSCs simultaneously down‐shifted the work function of the silver (Ag) and copper (Cu) electrodes and maintained good interfacial contact with the active layer. Consequently, a PCE of 17.2% was obtained from the OSCs composed of PM6:Y6 with PDINN as the cathode interlayer.^[^ ^17^ ^]^ PDI‐NO was synthesized as a cathode interlayer in indoor OSCs to facilitate charge extraction with lower charge carrier density. Owing to its deeper highest occupied molecular orbital level, PDI‐NO exhibited better hole‐blocking properties, leading to significantly lower leakage current and trap‐assisted recombination. As a result, the devices with PDI‐NO interlayer attained a PCE of 31% under 3000K LED light illumination.^[^ ^18^, ^19^ ^]^

Apart from the novel material designs, interface engineering with emerged cathode interlayers is a proven effective approach to enhance the cathode interlayer properties for high‐performance NFA OSCs.^[^ ^20^, ^21^, ^22^ ^]^ For example, an n‐n heterojunction composed of PNDITF3N and Phen‐NaDPO was used in OSCs composed of PM6:Y7, and a PCE of 17.1% was achieved, outperforming its single cathode interlayer counterparts. The enhanced performance was ascribed to the good film‐forming quality, combination of selective carrier transport properties, and reduced recombination.^[^ ^17^ ^]^ Besides, the variety of PNDIT‐F3N and PDINN was reported and used in the OSCs composed of PM6:Y6. Compared with the OSCs with PNDIT‐F3N, the PCE of the OSCs with hybrid cathode interlayer attained a 7.76% increase,^[^ ^23^ ^]^ resulting from enhanced charge extraction, better charge selectivity, and suppressed exciton recombination. However, only nitrogen (N)‐containing cathode interlayers have been selected to get the hybrid interlayer.^[^ ^20^, ^23^, ^24^ ^]^ The bromine (Br)‐containing interlayer materials, such as PFN‐Br and NDI‐NBr are rarely used in these combinations.^[^ ^25^ ^]^ Additionally, except for the impact of the hybrid cathode interlayer on the electrode work functions, the thin film quality, and charge recombination, how to rationally select the interlayer materials to get effective hybrid cathode interlayer lags.^[^ ^26^, ^27^, ^28^, ^29^ ^]^

In this work, we propose a double‐dipole strategy to enhance the properties of organic cathode interlayers, which is induced by incorporating N‐ and Br‐containing interlayer materials. To this end, we selected the state‐of‐the‐art active layer PM6:Y6 and fabricated conventional OSCs with Ag as the metal electrode. We chose three prototypical cathode interlayer material pairs with N and Br groups, PDIN: PFN‐Br, PDIN: NDI‐NBr, and PDINN: PFN‐Br, to evaluate the performance of such hybrid cathode interlayers. The Br ions tend to break from PFN‐Br and form a new chemical bond with the Ag electrode and could adsorb extra dipoles directed from the interlayer to Ag,^[^ ^30^ ^]^ which is in the same direction as the dipoles formed by the N‐containing interlayers. As a result, the double‐dipole could reduce the electrode work function, suppress the dark current leakage, and improve charge extractions, leading to enhanced short circuit current density (*J*_SC_) and fill factor (FF). Especially when the cathode interlayer PDIN: PFN‐Br (0.9:0.1, in wt.%) was used to fabricate OSCs composed of PM6:Y6, a champion PCE of 17.8% was attained. Our results provide guidelines on the selection of interlayer materials for high‐performance hybrid cathode interlayers in non‐fullerene OSCs.

## 2 Results and Discussion

**Figure 1:**
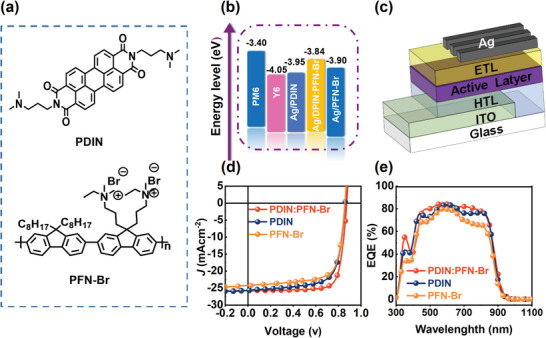
a) Chemical structures of the cathode interlayer materials. b) Energy levels of each functional layer. c) The schematic of the conventional device architecture. d) *J*–*V* characteristics and e) external quantum efficiency (EQE) spectra of the optimized devices.

**Figure** **1**a shows the chemical structures of the N and Br containing interlayer materials, PDIN and PFN‐Br, which we used as the models. The performance of the hybrid cathode interlayer was evaluated in OSCs comprising PM6: Y6, the state‐of‐the‐art active layer combination.^[^ ^31^, ^32^, ^33^, ^45^, ^46^ ^]^ The highest occupied molecular orbitals (HOMO) and lowest unoccupied molecular orbitals (LUMO) of PM6 and Y6 were taken from reported values (Figure 1b), and the rest were determined by ultraviolet photoelectron spectroscopy (UPS), which will be discussed in a later section. The ratio of PDIN and PFN‐Br was optimized by screening the PCE of the OSCs with varied cathode interlayer compositions, and detailed information is provided in the Supporting Information. To evaluate the device performance, we employed a conventional device architecture of ITO/PEDOT: PSS/PM6:Y6/cathode interlayer/Ag, as depicted in Figure 1c.

The *J*–*V* characteristics of the OSCs with PDIN, PFN‐Br, and PDIN: PFN‐Br (N‐Br) as cathode interlayer are plotted in Figure 1d. The devices with N‐Br exhibit dramatically enhanced FF and slightly increased *J*_SC_ than those with PDIN and PFN‐Br. The control devices, i.e., devices with PDIN as the cathode interlayer, exhibit an open‐circuit voltage (*V*_OC_) of 861 mV, a *J*_SC_ of 24.84 mA cm^−2^, and an FF of 72.9%, which leads to a PCE of 15.8%; whereas the devices with N‐Br attained a *V*_OC_ of 862 mV, a *J*_SC_ of 26.70 mA cm^−2^, and an FF of 73.8%, resulting in a PCE of 16.8%. Additionally, when 0.5% 1‐chloronaphthalene was added to the active layer, the devices with N‐Br got a champion PCE of 17.8%. In contrast, a modest PCE of 13.1% was obtained from the devices with PFN‐Br, associated with lower *J*_SC_ and FF. The photovoltaic parameters of all the devices are listed in **Table** **1**. When the solvent additive was used in the active layer, the PM6:Y6 devices with hybrid cathode interlayer achieved a champion PCE of 17.8% (17.5% averaged, Table S2, Supporting Information). The apparent changes in *J*_SC_ and FF could be ascribed to the electron‐rich interface caused by the accumulation of Br ions that broke from PFN‐Br and adsorbed at the Ag surface, significantly inhibiting the charge extractions from the devices. The changes in *J*_SC_ were also visualized in the external quantum efficiency (EQE) spectra of the devices, as shown in Figure 1e. In the wavelength range of 450–620 and 650–850 nm, the devices with N‐Br exhibit a stronger response than the control devices fabricated with PDIN, whereas the devices with PFN‐Br show the lowest EQE in this range.

**Table 1:** Photovoltaic parameters of PM6:Y6 OSCs with PDIN, PFN‐Br, and N‐Br hybrid cathode interlayers

PDIN: PFN‐Br [wt.%]	*V*_OC_ [mV]	*J*_SC_ [mA cm^−2^]	FF [%]	PCE^a)^ [%]
1:0	861 (855 ± 6)	25.1 (24.8 ± 0.2)	73.2 (72.8 ± 0.3)	15.8 (15.4 ± 0.3)
0.9:0.1	862 (860 ± 4)	26.7 (26.4 ± 0.2)	73.8 (73.4 ± 0.3)	16.7 (16.4 ± 0.2)
0.1	855 (852 ± 3)	24.9 (24.5 ± 0.4)	61.4 (60.6 ± 0.8)	13.1 (12.7 ± 0.2)

^a)^ Average values with standard deviation were obtained from 15 devices.

**Figure 2:**
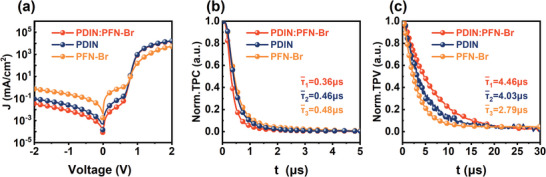
a) *J*–*V* curves of devices with PDIN, PFN‐Br, and N‐Br under the dark. b) Normalized transient photocurrent of devices with PDIN, PFN‐Br, and N‐Br. c) Normalized transient photovoltage of devices with PDIN, PFN‐Br, and N‐Br, recorded at open circuit conditions.

To check the impact of the cathode interlayer on the current leakage and charge extraction, we measured the *J*–*V* curves under the dark and the transient photocurrent (TPC). The dark *J*–*V* characteristics are displayed in **Figure** **2a** in the logarithm scale. The dark current density follows the following equation:^[^ ^44^ ^]^

(1) $$J\;=J_{0}\;\left[\exp\left(\frac{q\left(V-JR_{s}\right)}{\mathrm{nkT}}\right)-1\right]+\frac{V-JR_{s}}{R_{\mathrm{sh}}}$$

where n is the ideality factor, R_sh_ is the shunt resistance, R_s_ is the series resistance, and k is the Boltzmann constant. We fitted the dark *J*–*V* curves in the forward bias range to get the n, R_sh,_ and R_s_ (detailed in Figure S1, Supporting Information). The n values of 1.44, 2.06, and 1.47 were derived for the PDIN, PFN‐Br, and N‐Br devices, respectively. It was reported that *n* > 1 indicates the deviation from trap‐free conditions, and *n* = 2 implies the domination of trap‐assisted charge recombination. There could be interfacial traps between the active and cathode layer when different interlayers were used. Therefore, the control devices and OSCs with N‐Br suffer less extent of trap‐assisted charge recombination loss compared with the devices with PFN‐Br, which is in agreement with the FF obtained. The R_sh_ is a critical factor in inhibiting dark current leakage. The R_sh_ values of 7.6 × 10^4^, 4.8 × 10^3^, and 2.9 × 10^5^ Ω cm^2^ were obtained for the devices with PDIN, PFN‐Br, and N‐Br, respectively. As a result, the reverse saturated current density of the devices with N‐Br is the lowest compared with the PDIN and PFN‐Br counterparts, indicating significantly suppressed current leakage. The main impact of R_s_ is to reduce the FF, though excessive values may also lessen the J_SC_. The R_s_ of devices with PFN‐Br is one order higher than the other peers, which agrees with the lower FF attained.

Next, we utilized a mono‐exponential decay model to estimate the charge extraction information to fit the current decay measured via TPC.^[^ ^34^, ^35^, ^36^, ^37^ ^]^ The resulting extraction times were 0.36, 0.46, and 0.48 µs for devices with PDIN, PFN‐Br, and N‐Br, respectively, as shown in Figure 2b. These values were obtained by averaging three independent measurements, and the detailed fitting can be found in Figure S2 (Supporting Information). While there is a slight time difference of ≈0.1 µs, the time resolution is sufficient. Notably, the shorter extraction time observed in OSCs with N‐Br suggests that charges generated in these devices could be extracted more quickly than in the other two counterparts. Moreover, we performed the transient photovoltage measurements to get the charge carrier lifetime by fitting the transient voltage decay with a mono‐exponential decay model. The fitted lifetime of 4.03 µs for the control devices, 2.79 µs for the PFN‐Br devices, and 4.46 µs for the N‐Br devices were obtained (Figure 2c), indicating the interfacial traps are significantly reduced in the control and N‐Br devices.

**Figure 3:**
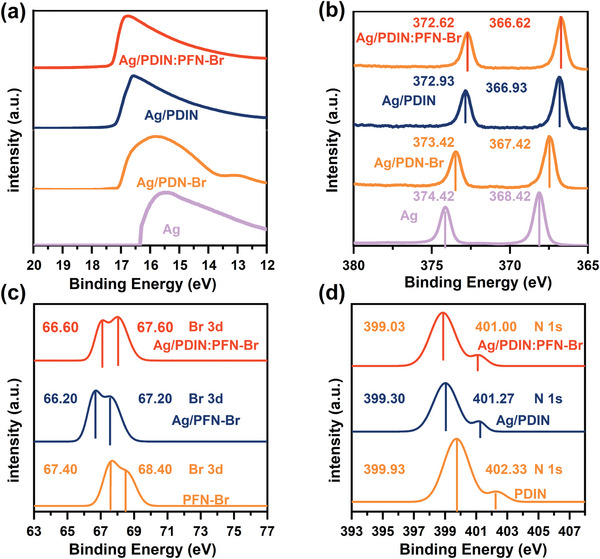
a) Ultraviolet photoelectron spectroscopy (UPS) spectra of the Ag electrodes covered with different cathode interlayers. b) X‐ray photoelectron spectroscopy (XPS) spectra of the electrodes: spectrum of Ag 3d on the pristine Ag, PFN‐Br/Ag, PDIN/Ag, and N‐Br/Ag surface. c) the spectrum of Br 3d on the PFN‐Br, PFN‐Br/Ag and N‐Br/Ag. d) the spectrum of N 1s on the PDIN, PDIN/Ag and N‐Br/Ag surfaces. The raw data are presented in Figure S5 (Supporting Information).

According to the integer charge transfer model, if the electrode work function (Fermi‐level) is higher than the acceptor's integer charge transfer states (E_ICT‐_), the Fermi‐level could be aligned with E_ICT‐_, leading to barrierless contact for charge extractions.^[^ ^20^, ^38^ ^]^ The work function of the electrode with and without cathode interlayers was measured with UPS, and the binding energy changes are presented in **Figure** **3**a. A work function of 4.5 eV is obtained for the bare Ag, consistent with previously reported values. When the cathode interlayer covers the Ag surface, the work function of the electrodes decreases, resulting in 3.90, 3.95, and 3.84 eV for PDIN/Ag, PFN‐Br/Ag, and N‐Br/Ag, respectively. As a result, all the electrodes could form an ohmic contact with the active layer, lowering the charge extraction barriers. However, the *V*_OC_ of the devices with PFN‐Br is slightly lower than those of the other two devices, owing to the electron‐rich surface caused by the accumulation of Br ions which will be discussed in the following section.

To confirm whether N and Br react with Ag in the N‐Br/Ag electrode, we performed X‐ray photoelectron spectroscopy (XPS) measurements, and the spectra are presented in Figure 3b–d. Compared with PDIN and PFN‐Br, the hybrid interlayer N‐Br is the most effective in reducing the binding energy of Ag from 366.6 to 372.2 eV, in agreement with the results of the UPS discussed earlier. Then, we aimed to confirm the physicochemical reaction between Br/Ag, N/Ag, and N‐Br/Ag. To this end, we prepared different surfaces and characterized them using XPS. In PFN‐Br films, the binding‐energy peaks of Br 3d are located at 68.4 and 67.4 eV. When we spin‐coated PFN‐Br onto the Ag surfaces, we observed a significant change in the binding energy peak, and the binding‐energy peaks of Br 3d are located at 67.2 and 66.2 eV, indicating the existence of Ag‐Br at the interface, as shown in Figure 3c (solid line at the bottom). This result is consistent with the calculation results demonstrated previously.^[^ ^25^ ^]^ Likewise, the binding energy peak of Br 3d for N‐Br films also changed, with peaks observed at 66.6 and 67.6 eV, as shown in Figure 3c (solid lines on top). We also investigated the binding‐energy peak of N 1s. In the pristine PDIN films, the N 1s peaks are observed at 399.9 and 402.3 eV, which align with the reported values of 400.0 and 402.0 eV for the N 1s in an organic compound. In PDIN/Ag films, the binding‐energy peaks of N 1s are found at 399.3 and 401.2 eV. However, in the N‐Br/Ag films, the binding‐energy peaks of N 1s shift to 399.0 and 401.0 eV. This change should be due to the Br ions breaking from PFN‐Br and forming a new chemical bond with the Ag electrode, which brings N closer to silver. Therefore, the binding‐energy peaks further reduce, as shown in Figure 3d (details in Figure S7, Supporting Information).

**Figure 4:**
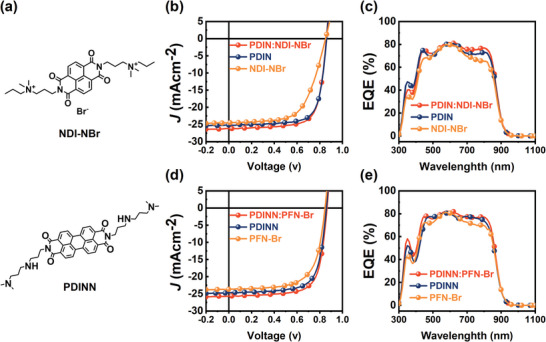
a) Chemical structures of the cathode interlayer materials. b) *J*–*V* characteristics and c) external quantum efficiency (EQE) spectra of the optimized devices with PDIN, NDI‐NBr, and N‐Br. d) *J*–*V* characteristics and e) external quantum efficiency (EQE) spectra of the optimized devices with PDINN, PFN‐Br, and N‐Br.

To assess the universality of the N‐Br hybrid cathode interlayer strategy, we fabricated OSCs with PDIN/NDI‐NBr and PDINN/PFN‐Br as the cathode interlayers. The chemical structures of NDI‐NBr and PDINN are presented in **Figure** **4**a. The optimized N‐Br ratio was identical to the model system PDIN/PFN‐Br, and the devices' performance with EQE spectra is plotted in Figure 4b–e. The PCE of the devices with FPN‐Br and NDI‐NBr is 13.0% and 13.5%, respectively, due to the Br ions causing electron extraction barriers at the Ag electrode. The PCE of the control devices is 15.8% and 15.0% for PDIN and PDINN, respectively. However, the PCE increased to 16.6% and 16.2% upon using the hybrid strategy, as summarized in **Table** **2**. The main contribution to the enhanced PCE was the improved *J*_SC_ and FF, resulting from the reduced charge extraction barrier and trap‐assisted recombination, as demonstrated above. The changes in *J*_SC_ are also visualized in the EQE spectra of the devices, and the significantly lower EQE response in the acceptor region implies poor electron extraction from the devices.

**Table 2:** Photovoltaic parameters of OSCs with PDIN, PDINN, NDI‐NBr, PFN‐Br, and the N‐Br hybrid cathode interlayers

Cathode interlayer	[wt.%]	*V*_OC_ [mV]	*J*_SC_ [mA cm^−2^]	FF [%]	PCE^b)^ [%]
PDINN: PFN‐Br	1:0	857 (854 ± 3)	24.6 (24.3 ± 0.3)	72.7 (71.4 ± 1.3)	15.1 (14.8 ± 0.3)
	0.9:0.1	861 (857 ± 4)	26.7 (26.2 ± 0.5)	72.6 (71.8 ± 0.7)	16.0 (15.7 ± 0.2)
	0:1^a)^	853 (850 ± 3)	24.9 (24.5 ± 0.4)	61.4 (60.6 ± 0.8)	13.1 (12.7 ± 0.2)
PDIN:NDI‐NBr	1:0^a)^	860 (856 ± 4)	25.1 (24.8 ± 0.2)	73.2 (72.8 ± 0.3)	15.8 (15.4 ± 0.3)
	0.9:0.1	863 (858 ± 5)	26.1 (26.2 ± 0.2)	74.54 (73.7 ± 0.7)	16.6 (16.3 ± 0.2)
	0:1	852 (849 ± 3)	23.8 (23.4 ± 0.4)	67.60 (67.2 ± 0.3)	13.5 (12.7 ± 0.2)

^a)^ The performance is identical with the devices presented in Table 1 owing to the identical device architecture and materials used;

^b)^ Average values with standard deviation were obtained from 15 devices.

**Figure 5:**
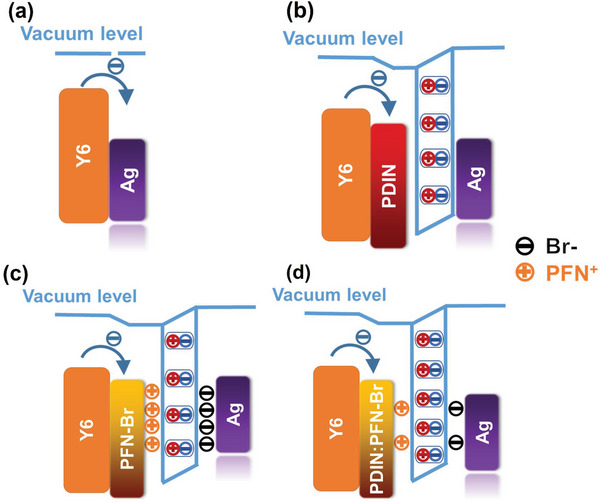
Diagram of the electron extraction from devices a) without cathode interlayer, b) with N‐containing cathode interlayer, c) with Br‐containing cathode interlayer, d) with N‐Br containing cathode interlayer.

Previous reports have suggested that the ammonium group is crucial in reducing the metal electrodes' work function.^[^ ^39^, ^40^, ^41^ ^]^ However, recently N‐ and Br‐containing groups were also demonstrated to reduce the Ag work function effectively, changing from 4.46 to 4.22 eV and 4.46 to 4.06 eV,^[^ ^24^, ^25^ ^]^ respectively. Especially, the Br ions tend to break from FPy‐Br and form a new chemical bond with Ag, which could adsorb extra dipoles directed from the interlayer to Ag and lead to the formation of a dense layer of electronegative Br ions at the metal surface.^[^ ^13^ ^]^ **Figure** **5** schematics illustrate the impact of cathode interlayers. Figure 5a shows that when there is no electron transport layer, the active layer is in direct contact with the cathode, which leads to a considerable loss in electron extraction due to the fermi‐level pinning, resulting in poor device efficiency and stability.^[^ ^42^, ^43^ ^]^ When PDIN is used as the cathode interlayer, the electric dipoles pointing to Ag could form, facilitating charge extraction (Figure 5b). While PFN‐Br or NDI‐NBr, the Br‐containing layer, is employed, dipoles resulting from the Br ions on the Ag surface could form, as depicted in Figure 5c. However, the electron‐rich Ag surface could negatively affect the charge extraction from devices, severely depressing the electron collection efficiency, which may be the main reason for the lower *J*_SC_ and FF in the devices with PFN‐Br or NDI‐NBr. Finally, Figure 5d presents the double dipole conditions. First, PDIN is the major component in the cathode interlayer, and the nitrogen group‐caused dipoles are effective. Second, the minority composition of the Br‐containing interlayer leads to extra dipoles directed from the interlayer to Ag due to the abovementioned reasons, but with significantly fewer Br ions on the Ag surface. As a result, the double dipoles could strengthen the built‐in electric field, suppress the dark current leakage, and improve charge extractions, leading to enhanced *J*_SC_ and FF.

## 3 Conclusion

In summary, we proposed a double‐dipole strategy to enhance the properties of organic cathode interlayers by incorporating N‐ and Br‐containing interlayer materials. The impact of the double‐dipole on the device performance and electrode modification was investigated using OSCs composed of PM6:Y6, which are known for their state‐of‐the‐art performance. We demonstrated that adding 10% Br‐containing materials to the PDIN or PDINN host resulted in N‐Br hybrid cathode interlayers that achieved higher FF and *J*_SC_ due to reduced interfacial traps and charge extraction barriers and led to a champion PCE of 17.8%. The hybrid cathode interlayer offers several advantages, including lowered electrode work functions, suppressed dark current leakage, and improved charge extractions. Our results provide a novel hybrid cathode interlayer construction strategy and valuable insights into the selection of interlayer materials for high‐performance nonfullerene OSCs.

## Conflict of Interest

The authors declare no conflict of interest.

## Supporting information

Supporting Information

## Data Availability

The data that support the findings of this study are available in the supplementary material of this article.
