# Supplementary material for: Double‐Dipole Induced by Incorporating Nitrogen‐Bromine Hybrid Cathode Interlayers Leads to Suppressed Current Leakage and Enhanced Charge Extraction in Non‐Fullerene Organic Solar Cells
Source: Adv Sci (Weinh). 2023 Jul 3;10(26):2302460. doi: 10.1002/advs.202302460 (PMC10502809; doi:10.1002/advs.202302460)
Supplement: Supplementary file 1 — Supporting Information [file ADVS-10-2302460-s001.pdf]

ADVANCED SCIENCE Open Access

Supporting Information

for *Adv. Sci.*, DOI 10.1002/advs.202302460

Double-Dipole Induced by Incorporating Nitrogen-Bromine Hybrid Cathode Interlayers Leads to Suppressed Current Leakage and Enhanced Charge Extraction in Non-Fullerene Organic Solar Cells

*Yangchao Zheng, Jingjing Zhao, Huanpeng Liang, Zhenmin Zhao and Zhipeng Kan**

Supporting Information

Double-Dipole Induced by Incorporating Nitrogen-Bromine Hybrid Cathode Interlayers Leads to Suppressed Current Leakage and Enhanced Charge Extraction in Nonfullerene Organic Solar Cells

*Yangchao Zheng, Jingjing Zhao, Huanpeng Liang, Zhenmin Zhao, Zhipeng Kan**

Y. Zheng, J. Zhao, H. Liang, Z. Zhao, Prof. Z. Kan
Center on Nano Energy Research, Guangxi Colleges and Universities Key Laboratory of Blue Energy and Systems Integration, Carbon Peak and Neutrality Science and Technology Development Institute, School of Physical Science & Technology, Guangxi University, Nanning 530004, China
Email: kanzhipeng@gxu.edu.cn
Prof. Z. Kan
State Key Laboratory of Featured Metal Materials and Life-cycle Safety for Composite Structures, Nanning 530004, China

Content

1. Materials 2
2. Device Fabrication 2
3. Additional PV Device Performance Data 2
4. Transient Photocurrent and Transient Photovoltage (TPC and TPV) 4
5. UPS and XPS 5

1. Materials

PBDBT-2F (PM6), Y6, PDIN, PDINN, PFN-Br and NDI-NBr were purchased from Solarmer, Inc (Beijing). All materials were used as received without further purification.

2. Device Fabrication

OSC devices were fabricated using indium tin oxide (ITO)-coated glass substrates, which were cleaned with diluted detergent, deionized water, acetone and isopropyl alcohol in an ultrasonic bath sequentially for 30 min, and further treated for 30 min in a UV ozone chamber.

The BHJ structures was ITO/PEDOT:PSS/PM6/Y6/ETL/Ag. The blend solutions were prepared by dissolving PM6 and Y6 in chloroform solvent with 1-CN content 0.5%. The total concentration of all active layer solutions was maintained at 16 mg/ml. The active layers were generated by spin-coating the blend solutions (the volume used per round is 14μL) at a spin-coating rate of 4500 r.p.m. for 30 s on the top of PEDOT:PSS with an optimal thickness of 105 nm .A thin layer (~5nm) of PDIN(2mg/ml),PFN-Br(0.5mg/ml) or the mix concentration that PDIN:PFN-Br (0.9:0.1, in wt%) of 2mg/ml was spin-cast on the top of the active layer at a spin-coating rate of 5,000 r.p.m. for 30 s. Finally, a 100-nm-thick Ag electrode was thermally deposited under vacuum conditions of 2×10^{-6}Pa.

The current density-voltage (J-V) curves of devices were measured using a Keithley 2400 Source Meter in glove box under AM 1.5G (100 mW cm^{-2}) using a Enlitech solar simulator. The EQE spectra were measured using a Solar Cell Spectral Response Measurement System QE-R3011 (Enlitech Co., Ltd.). The light intensity at each wavelength was calibrated using a standard monocrystalline Si photovoltaic cell. The temperature dependent J-V and CELIV were measured with PAIOS platform with low temperature unit. (Fluxim, Swizerland)

3. Additional PV Device Performance Data

Table S1. Photovoltaic performance of PM6:Y6 based solar cells with different PDIN:PFN-Br ratio.

PDIN：PFN-Br	V_{OC} (V)	J_{SC} (mA/cm^2)	FF (%)	PCE (%)	**Ave.PCE^a (%)**
1:0	856	25.1	72.6	15.8	15.6
0.95:0.05	862	26.4	72.9	16.6	16.4
0.9:0.1	864	26.7	73.8	16.7	16.5
0.8:0.2	857	26.0	72.5	16.2	16.0

0.7:0.3	853	25.6	71.2	15.7	15.5
0:1	849	24.9	61.4	13.0	12.7

[a]Average values with standard deviation were obtained from 15 devices.

Table S2. Photovoltaic performance of PM6:Y6 based on PDIN:PFN-Br with 1-CN content 0.5%

PDIN : PFN-Br (0.5% 1-CN)	V_{OC} (V)	J_{SC} (mA/cm^2)	FF (%)	PCE (%)	**Ave.PCE[a] (%)**
1:0	863	25.6	77.1	17.1	16.8
0.9:0.1	872	25.8	78.9	17.8	17.5
0:1	852	24.6	68.7	14.4	14.1

[a]Average values with standard deviation were obtained from 15 devices.

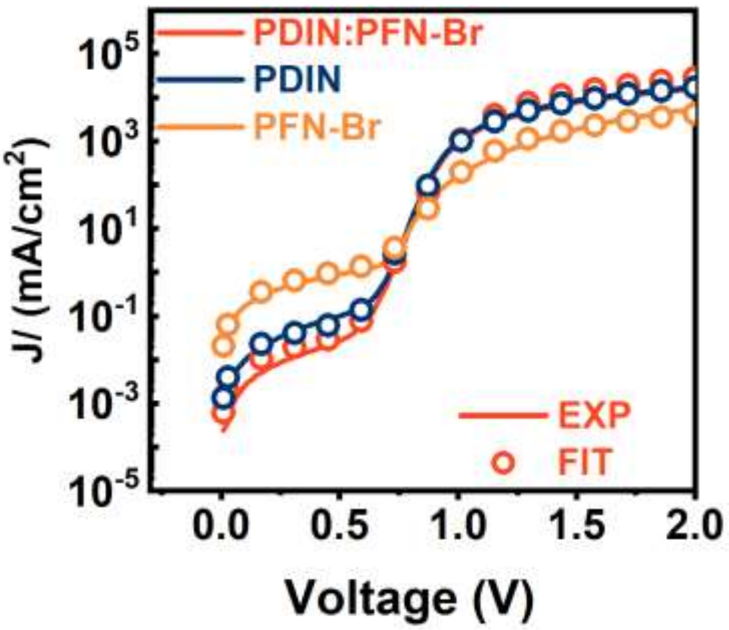

Figure S1. J-V curves of devices with PDIN, PFN-Br, and N-Br under the dark. The solid lines represent the experimental data, while the open symbols were obtained by fitting the experimental data to the diode equation.

Table S3. fitting the experimental data of devices with PDIN, PFN-Br, and N-Br under the dark

Cathode interlayer	n	Rs (Ohm m^2)	Rsh (Ohm m^2)
PDIN:PFN-Br	1.475±0.019	$6.108\times10^{-5}\pm2.414\times10^{-6}$	29.687±1.382

PFN-Br	2.060±0.089	$2.081\times10^{-4}\pm1.034\times10^{-5}$	0.485±0.009
PDIN	1.445±0.019	$5.676\times10^{-5}\pm2.083\times10^{-6}$	7.632±0.317

4. Transient Photocurrent and Transient Photovoltage (TPC and TPV)

For TPV, the measurement was conducted under 1 sun condition by illuminating the device with a white light-emitting diode, and the champion device is set to the open-circuit condition. For TPC, the champion device is set to the short-circuit condition in dark. The output signal was collected by key sight oscilloscope. The transient photocurrent (TPC) was tested under the short-circuit condition to explore the time-dependent extraction of photogenerated charge carriers. The 10 ns light plus laser were selected as the light source for steady the photogenerated current density. The devices are otherwise kept in the dark between pulsesto avoid any influence of pulse frequency on the current responses. The transient photovoltage (TPV) was tested under the open-circuit condition to explore the photovoltage decay.

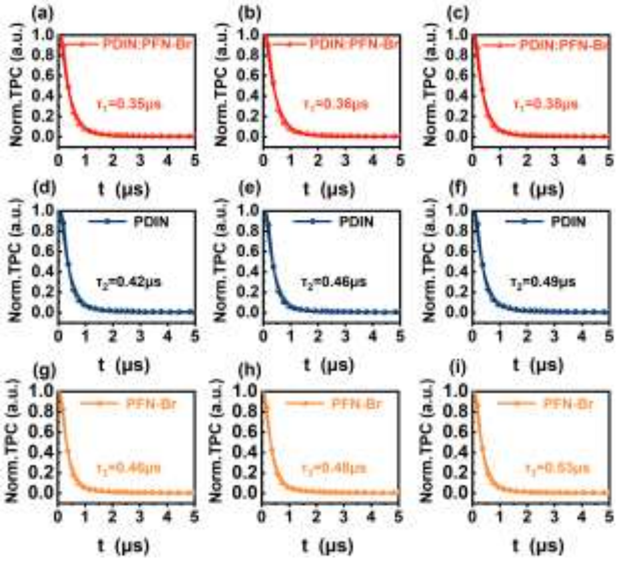

Figure S2 . Normalized transient photocurrent of devices with PDIN, PFN-Br, and N-Br

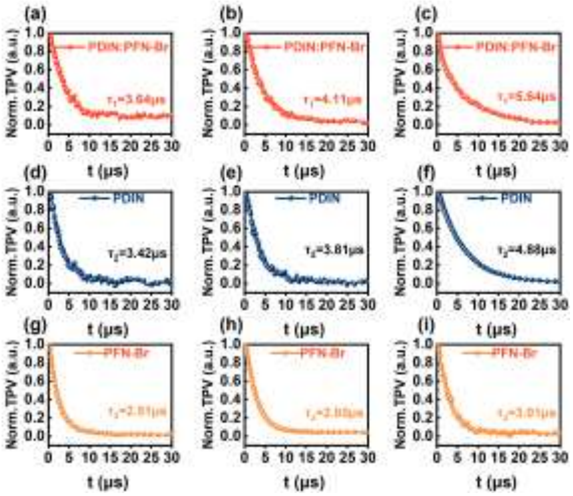

Figure S3. Normalized transient photovoltage of devices with PDIN, PFN-Br, and N-Br

5. UPS and XPS

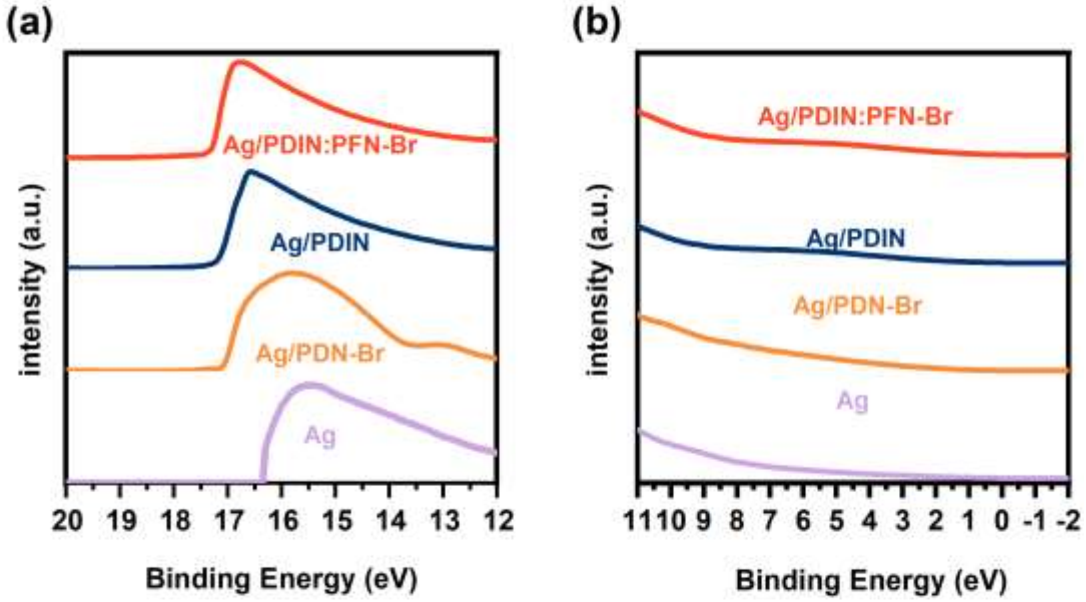

Figure S4. (a-b) Ultraviolet photoelectron spectroscopy (UPS) spectra of the Ag electrodes covered with different cathode interlayers

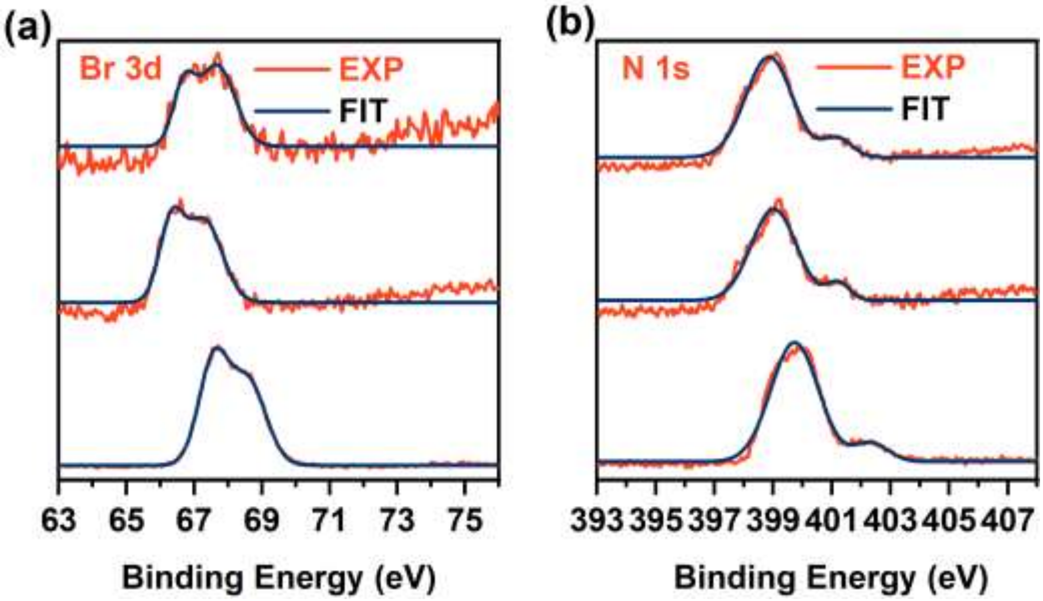

Figure S5. (a) Spectrum of Br 3d on the PFN-Br, PFN-Br/Ag and N-Br/Ag. (b) Spectrum of N1s on the PDIN, PDIN/Ag and N-Br/Ag surface. The red lines are experiment raw data, and the green lines are fitting peaks.
